# Cortical contributions to locomotor primitives in toddlers and adults

**DOI:** 10.1016/j.isci.2022.105229

**Published:** 2022-09-28

**Authors:** Coen S. Zandvoort, Andreas Daffertshofer, Nadia Dominici

**Affiliations:** 1 Department of Human Movement Sciences, Faculty of Behavioural and Movement Sciences, Amsterdam Movement Sciences & Institute for Brain and Behavior Amsterdam, Vrije Universiteit Amsterdam, Amsterdam 1081BT, the Netherlands

**Keywords:** Behavioral neuroscience, Biological sciences, Neuroscience

## Abstract

The neural locomotor system strongly relies on spinal circuitries. Yet, the control of bipedal gait is accompanied by activity in motor cortex. In human gait control, the functional interaction between these cortical contributions and their spinal counterparts are largely elusive. We focused on four spinal activation patterns during walking and explored their cortical signatures in toddlers and adults. In both groups, cortico-spinal coherence analysis revealed activity in primary motor cortex to be closely related to two of the four spinal patterns. Their corresponding muscle synergies are known to develop around the onset of independent walking. By hypothesis, the cortex hence contributes to the emergence of these synergies. In contrast, the other two spinal patterns investigated here resembled those present during newborn stepping. As expected, they did not show any cortical involvement. Together, our findings suggest a crucial role of motor cortex for independent walking in humans.

## Highlights

• Walking toddlers and adults show cortico-synergy coherence in the motor cortex
• The motor cortex mainly interacts with two out of four synergies
• Cortico-synergy coherence changes between toddlers and adults

## Graphical abstract

**Figure:**
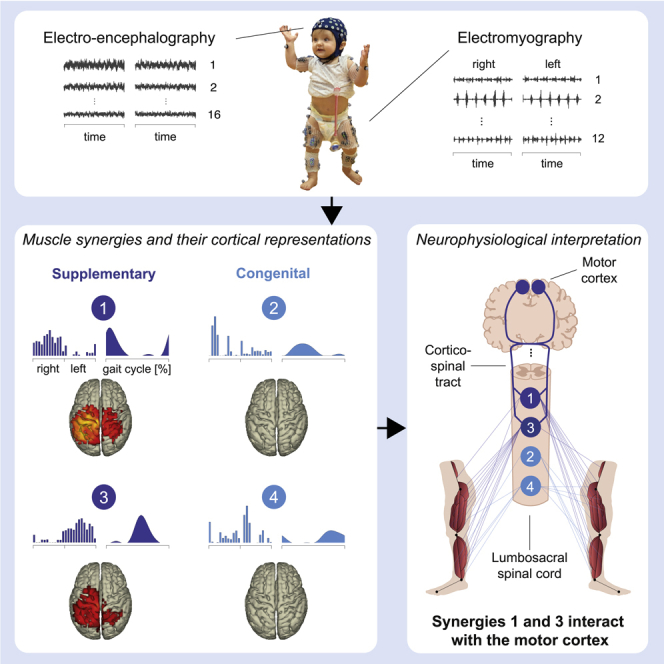

## Introduction

Walking involves spinal networks that accommodate locomotor primitives (Dominici et al., 2011; Grillner, 1985, 2006; Ivanenko et al., 2013). In vertebrate models, the spinal networks have been identified as populations of premotor interneurons located in the lumbosacral spinal cord and are often considered central pattern generators (CPGs) (Giszter, 2015; Takei et al., 2017). Locomotor primitives (or muscle synergies) comprise small sets of functional units generating the motor output to coordinate muscles (Dominici et al., 2011). Each of the primitives reduces neural dimensionality by imposing fixed activation patterns on a set of muscles (d'Avella et al., 2003). The activation patterns appear at distinct timings throughout the step cycle as shown for both animals and humans (Dominici et al., 2011). While the representation of muscular patterns via synergies appears established (Dominici et al., 2011), it is unclear how they are organized across the human nervous system (Bizzi and Cheung, 2013).

In humans, deciphering the neural basis of muscle synergies is a challenge. It typically capitalizes on nervous system manipulations or developmental courses (Cheung et al., 2020; Cheung and Seki, 2021; Dominici et al., 2011). Early locomotor development can be characterized by the preservation of congenital spinal patterns and superimposition of emerging patterns (Dominici et al., 2011; Sylos-Labini et al., 2020). In newborn stepping, two locomotor primitives/muscle synergies can be observed (Dominici et al., 2011), which alternately activate flexor and extensor muscles to move the legs (Thelen and Cooke, 1987). Constant time delays between the foot/ground contact and the activity of leg muscles imply immature neonate stepping to be driven by segmental reflex pathways (Forssberg, 1985). Similarities in stepping between typically developing and anencephalic infants further suggest that these neural circuitries are organized at or below the brainstem (Peiper, 1963). Cortical motor areas and cortico-spinal tracts around this age are immature as the motor system must still acquire most of its myelin (Yang and Gorassini, 2006). One may conjecture that the “hard-wired” neural circuitries governing locomotor primitives to be pattern generating networks that are separated from descending brain input.

Independent walking in toddlers coincides with the emergence of two additional muscle synergies on top of the two congenital ones of newborn stepping (Dominici et al., 2011). Along the aforementioned conjecture, these two sets with two synergies each are likely to rely on distinct phylogenetically neural systems (Forssberg, 1985). Expectably, the new set emerges after necessary structural and functional re-organizations of intra-spinal, sensory, and supra-spinal pathways (Forssberg, 1999). Regarding the latter synergy set, we here investigate the involvement of cortical motor networks. The motor cortex — much like the cortico-spinal tract — develops significantly over the first two years after birth (Eyre et al., 1991, 2000; Huttenlocher, 1979), presumably caused by myelin build-up (Kinney et al., 1988).

Many mammals can walk without neural input from supra-spinal areas by capitalizing solely on spinal CPGs (Forssberg et al., 1980; Grillner and Zangger, 1979), at least, if obstacle avoidance is not required (Drew et al., 2008). In quadrupeds, four locomotor primitives can be generated throughout the gait cycle without supra-spinal input (Rossignol et al., 2006). All the four primitives remain largely intact in rodents that acquired a lesion during neonatal and adult phases (Yang et al., 2019). This is remarkable since cortical areas project to the midbrain, brainstem, and spinal cord — all these pathways appear evolutionary stable (Ocaña et al., 2015). In primates, however, interrupting pyramidal tracts or descending brainstem pathways does yield impaired locomotion (Lawrence and Kuypers, 1968a; b), arguably, because there, the cortico-spinal tract does contain monosynaptic pyramidal projections to leg muscles (Lemon, 2008).

Bipedal locomotion involves motor centers in the brain (Nielsen, 2003). Especially, human gait is irrevocably affected without input from supra-spinal motor projections (Capaday, 2002; Oudega and Perez, 2012) and a complete interference at the spinal cord will cause an inability to walk (Dimitrijevic et al., 1998). Intact supra-spinal areas and a (largely) complete cortico-spinal tract are hence prerequisite for self-sustained walking in humans.

We studied the involvement of the cortico-spinal tract in the control of human gait by focusing on four spinal motor patterns during locomotion (Dominici et al., 2011; Grillner, 1985, 2006; Ivanenko et al., 2013). We expected cortical activity to be not coherent with the two congenital locomotor synergies whereas it should be coherent with the two synergies that are known to emerge with independent walking in toddlers. As such, functional neural re-organizations including plasticity in both motor cortex and cortico-spinal tract may contribute to establishing these synergies.

## Results

**Figure 1:**
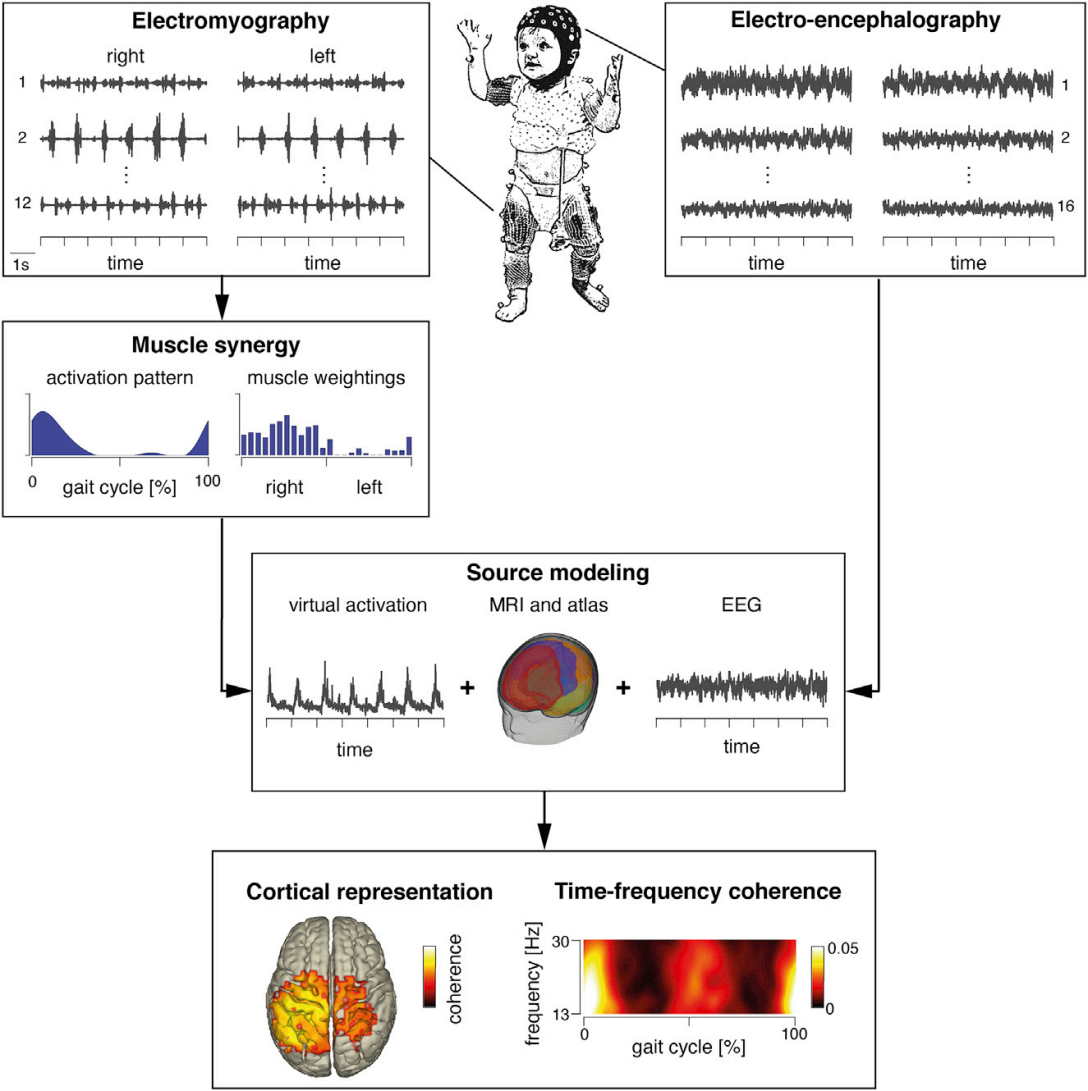
Experimental and methodological workflow We recorded 24-channel electromyography (EMG) and 32-channel electro-encephalography (EEG) during walking (top left and right, respectively). EMG signals of bilateral trunk and leg muscles were decomposed into muscle synergies (in dark blue an example of a temporal activation pattern and muscle weightings). Then, both (EMG and muscle weightings) were exploited to estimate virtual activation patterns (see body text for more details). When source modeling, cortical coherence representations between the virtual activation patterns and EEG were localized using beamforming and age-specific anatomical MRIs. Signal reconstruction allowed for estimating coherence between synergies and primary motor cortex as a function of the gait cycle and frequency.

To test for the hypothesized segregation of locomotor primitives into those with and without sole sub-cortical organization and the maturation of the latter, we analyzed correlative neural outputs from the cortex and the muscles that we recorded using non-invasive electrophysiological modalities during walking. We studied two groups — toddlers and adults — in which all four primitives were established (Dominici et al., 2011). In both groups, we co-registered electro-encephalographic (EEG) activity and electromyographic (EMG) activity of trunk and leg muscles (Figure 1). Cortical muscle synergy signatures were assessed by combining conventional muscle synergy and coherence-based source modeling analyses (d'Avella et al., 2003; Groβ et al., 2001). In brief, cortico-synergy coherence served as a measure of long-distance phase locking between cortical areas and muscle synergies (Zandvoort et al., 2019, 2021). We specifically targeted neural synchronization of the cortico-spinal drive in the beta-frequency band (13–30 Hz) as these oscillations are known to contribute to cortical control of voluntary movements (Baker, 2007; Engel and Fries, 2010; Kilner et al., 2000), locomotion (Roeder et al., 2018; Zandvoort et al., 2021), and postural control (Zandvoort et al., 2019).

### Muscle synergies and coherence-based beamformers

**Figure 2:**
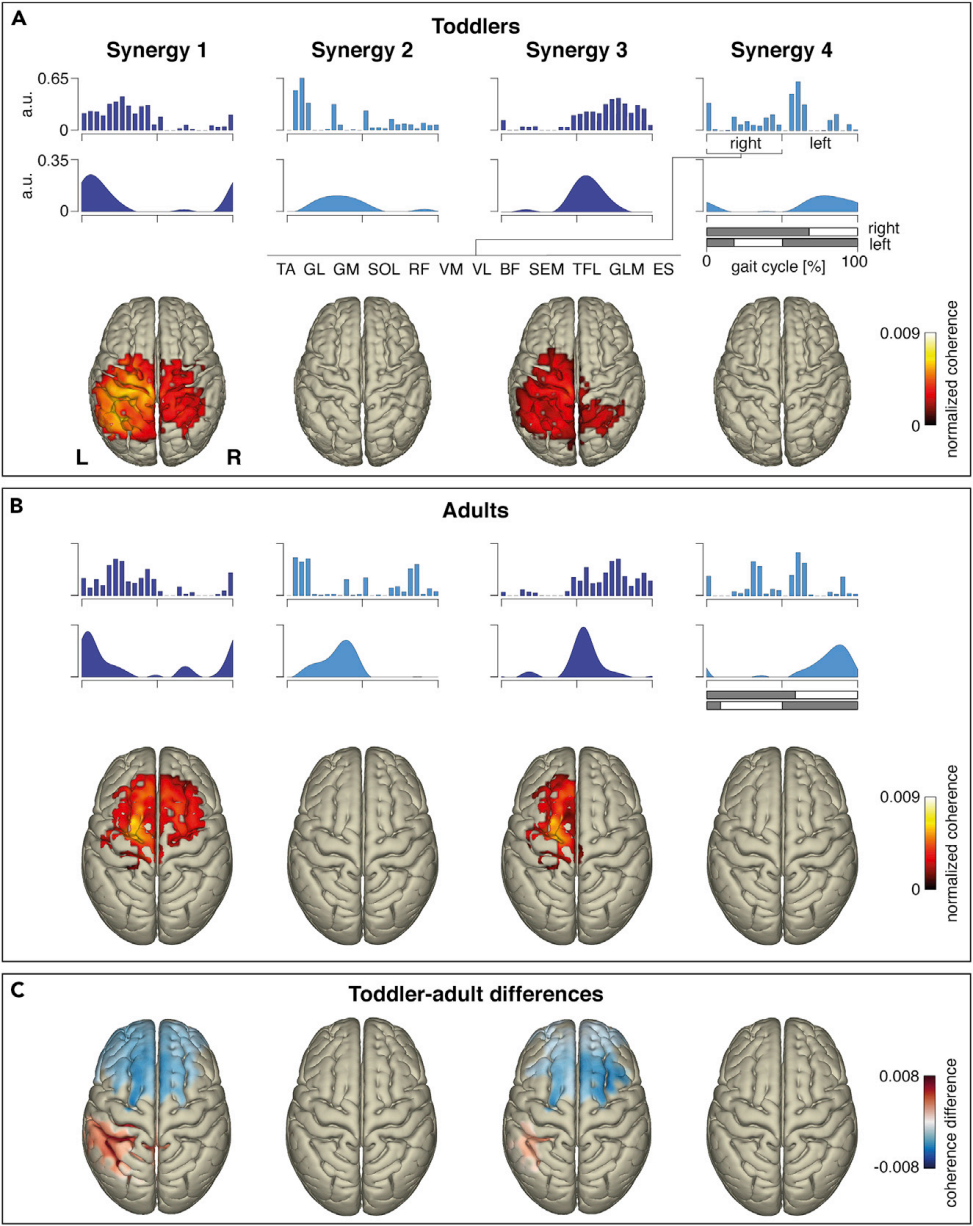
Muscle synergies and their cortical coherence representations for (A) toddlers, (B) adults, and (C) toddler-adult differences (A and B) Blue-colored bars and areas show ensemble-averaged muscle weightings and activation patterns. The latter are time locked from right-to-right foot contact (percentage of the gait cycle). TA, tibialis anterior; GL, gastrocnemius lateralis; GM, gastrocnemius medialis; SOL, soleus; RF, rectus femoris; VM, vastus medialis; VL, vastus lateralis; BF, biceps femoris; SEM, semitendinosus; TFL, tensor fasciae latae; GLM, gluteus maximus; ES, erector spinae. Dark and light blue colors refer to the group of synergies 1 and 3, and synergies 2 and 4, respectively. Reconstructed coherence representations between electro-encephalography and muscle synergies are projected onto age-matched cortical surfaces. Colored parts on these surfaces correspond to the significant voxels determined via one-sided, intra-group t-tests (p < 0.005; see Figure S3 for the statistical assessments) and indicate areas of the cerebral cortex at which coherence exceeds the individual subjects’ means. (C) Cortical surfaces demonstrated significant mean coherence differences as obtained by the inter-group independent t-tests, i.e., contrasting toddlers versus adults. Positive and negative values, hence, correspond to “toddlers > adults” and “toddlers < adults” (p < 0.025; see Figure S9 for the statistical assessments). See also Figures S1–S10 and Tables S1 and S2. L: left; R: right.

For the synergy assessments, we relied on the linear decomposition of muscular activation profiles through non-negative matrix factorization (Figure S1). Both toddlers and adults displayed four locomotor primitives (Figures 2A and 2B). Synergy patterns 1 and 3 were mainly active during double support phases, seemingly involved in the touch-down and lift-off of the legs. Synergy patterns 2 and 4 occurred during single support phases contributing to forward propulsion and swinging the legs. The temporal shapes of synergies 1 and 3 largely corresponded to the supplementary primitives reported in earlier work, and the ones for synergies 2 and 4 were comparable to the congenital primitives (Dominici et al., 2011). The cortical sensorimotor, supplementary motor, and premotor areas displayed significant beta-band coherence sources for synergies 1 and 3 (Figures 2A, 2B, and S2–S7). Cortical sources were absent for synergies 2 and 4. That is, only two out of four muscle synergies displayed significant cortical representations and that was evident in both toddlers and adults. Yet, the beta-band coherences appeared more posterior for toddlers compared to adults (Figures 2A and 2B). Beta-band coherences of synergies 1 and 3 showed a significant increase and decrease in premotor areas and somatosensory areas, respectively, suggesting further development and maturation from toddlers to adults (Figures 2C and S8–S10).

### Time-frequency coherence

**Figure 3:**
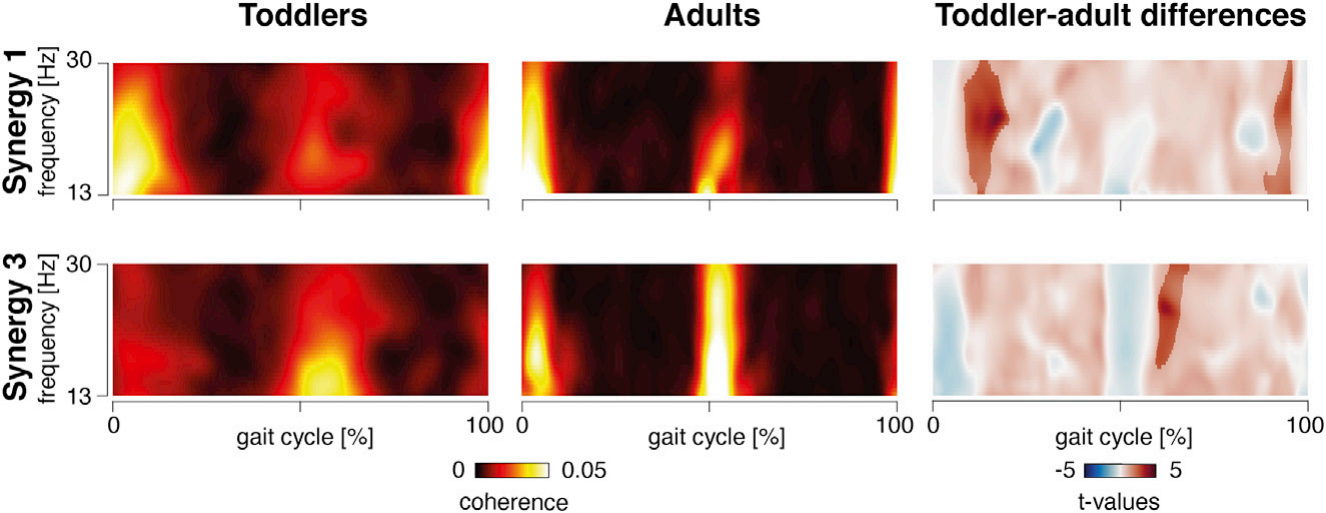
Time-frequency magnitude-squared coherence between the primary motor cortex and synergies 1 and 3 for toddlers and adults The gait cycle is defined from right-to-right foot contact. Time-frequency coherences are pooled over participants for every synergy pattern and group. For the toddler-adult differences, time-frequency statistics were estimated with cluster-based permutation tests using independent t-tests. Non-transparent colors correspond to statistically significant clusters. The positive t-values mean higher coherence for toddlers compared to adults. See also Figures S11–S14.

The time-resolved beta-band coherence between the source-reconstructed activity of primary motor areas and synergy patterns 1 and 3 was maximal during double support phases (Figures 3, S11, and S12), i.e., when the peak amplitude of the synergy patterns can be observed (Figures 2A and 2B). This reveals that coherence and synergy patterns were temporally aligned. Such a time locking can also be established between the beta-power modulation and virtual activation patterns (Figure S13). Coherence at double support phases is also evident for the cortico-muscular coherence estimates (Figure S14). Around the double support phase, the coherences involving synergies 1 and 3 appeared higher in the toddlers than in the adults, indicating that coherent epochs became more focal in time in the adults. For synergy 1, this was significant after the right heel contact, for synergy 3 after the left one (see Figure 3, right panels). They differed between groups in that coherence epochs became more focal in the adults. That is, while beta-band coherence is already present in toddlers, the cortico-spinal pathways carrying beta-band oscillations mature until adulthood (Figure 3).

## Discussion

**Figure 4:**
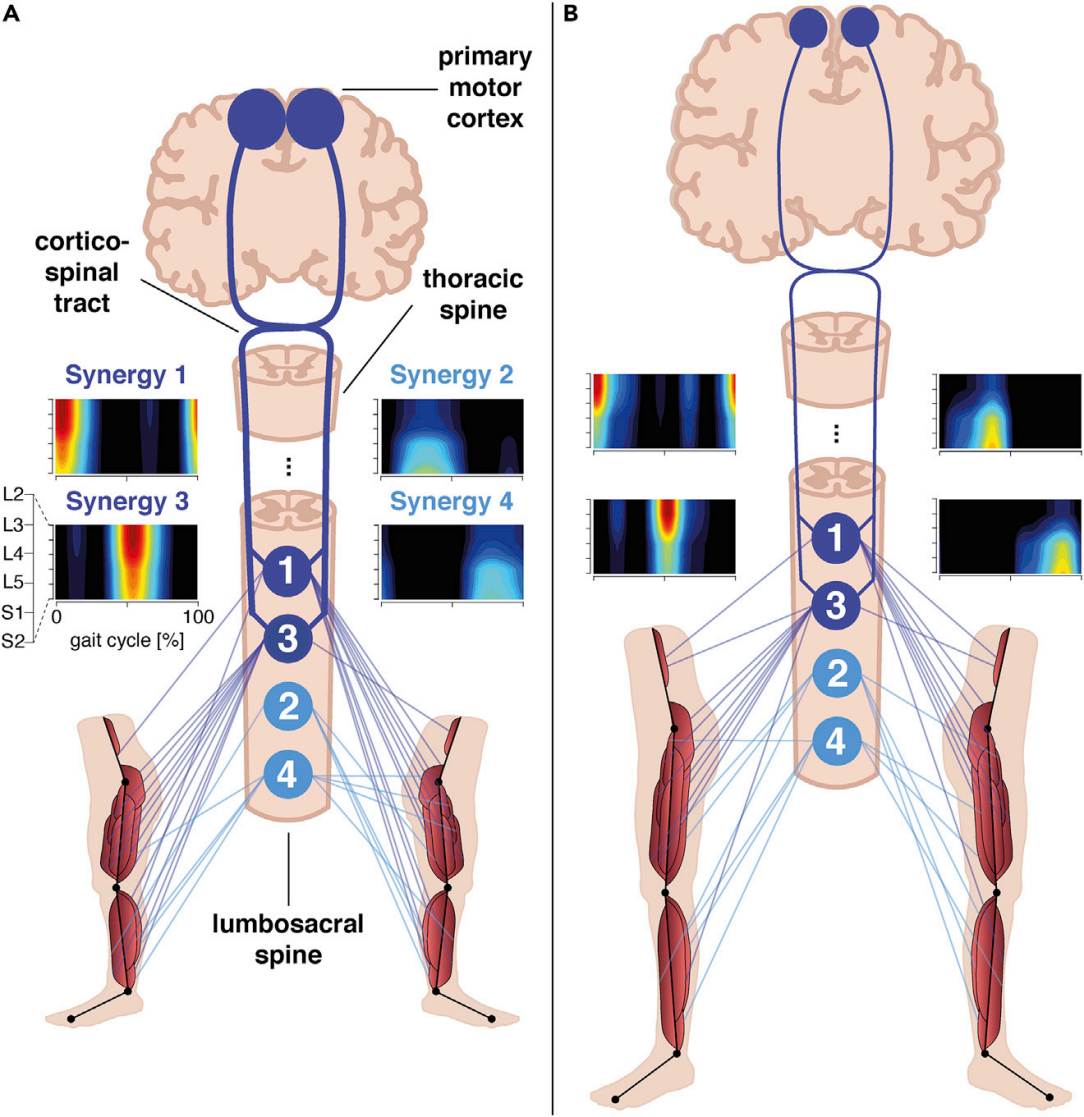
Conceptualization of how muscle synergies relate to activity in the primary motor cortex in (A) toddlers and (B) adults The four locomotor primitives are represented at the lumbosacral spinal cord as pinpointed by the spinal maps (i.e., spatiotemporal reconstructions of synergy activity at the spinal cord) ranging from segments L2 to S2 (Ivanenko et al., 2013; Wenger et al., 2016). As confirmed by the spinal maps, synergies 1 and 3 are accommodated more lumbar in the spinal cord compared to synergies 2 and 4. Every synergy co-activates multiple muscles with a specific weight (dark and light blue lines from the 1 to 4 synergies to the corresponding muscles – muscle weightings>0.15). The two dark-blue synergies — circles “1” and “3” — reveal phase-locking with oscillations in motor areas. This, however, does not necessarily mean that these synergies solely rely on activities in motor cortex. Yet, from our findings, we can only infer cortico-spinal interactions. Admittedly, this gross schematic lacks lots of detail, e.g., the involvement of cerebellum and deeper cerebellar nuclei like thalamus and basal ganglia (Berger et al., 2020; Cheung and Seki, 2021; Forssberg, 1985). No cortico-spinal pathways appear in the two light-blue synergies — circles “2” and “4” — which must be regulated by sub-cortical circuitries. The bigger cortical sources and thicker cortico-spinal tract in toddlers indicate stronger coherence between synergies and primary motor cortex (Figure 2C), and broader beta-band bursts in the time-frequency coherence (Figure 3), respectively.

The cortical representations and time-dependent coherences reported here provide first support that in humans two locomotor primitives relate to cortical areas. We translated these findings into a schematic shown in Figure 4, where we highlighted the cortex-dependent motor primitives by discarding any additional neural circuitries related to, e.g., cerebellum, thalamus, and basal ganglia (Berger et al., 2020; Cheung and Seki, 2021; Forssberg, 1985) for their contribution to the control of motor primitives.

We found synergies 1 and 3 to be represented in motor network activity both in toddlers and adults. The corresponding cortico-synergy coherence was most pronounced during the double support phases. This particularly applies to oscillatory activity in the beta-frequency band that seemingly propagates along the cortico-spinal tract to neuronal layers in the spinal cord (Baker, 2007). Its presence around the double support phases suggests cortical control of (dorsiflexor) leg muscles involved in the touchdown of the foot. Neonatal stepping typically does not display a clear touchdown and — remarkably —- this age group lacks synergies 1 and 3. Like quadrupeds, neonates touch the ground with their forefoot after which they lower their ankle through dorsiflexion, indicating a lack of ankle control (Forssberg, 1985). Here, we would like to note that leg muscle activity can be suppressed before foot contact by stimulating the motor cortex (Petersen et al., 2001). In any case, our findings hint at an important role of motor cortex in the activation of the lower leg muscles around foot contact. By contrast, synergies 2 and 4 seem to utilize sub-cortical structures for control, e.g., deeper cerebellar, brain stem, and spinal networks. They were not fostered by cortical networks and may stem from pattern generators that are (largely) independent of cortical input.

Cortical signatures of synergies 1 and 3 develop further from toddlers to adults (Figures 2C and 3). In the cerebral cortex, the muscle synergy signatures seem to shift from the primary somatosensory and motor areas in toddlers to premotor areas in adults (Figure 2C). In the early stages of walking development, they may therefore reflect sensory rather than motor processes. Recall that synergies 1 and 3 were primarily active during double support phases, when both legs are in contact with the ground calling for balance control via tangential forces decelerating and accelerating the body (Dominici et al., 2011). Around the double support phases, cortico-synergy coherence becomes more focal in time in the adults in agreement with earlier findings on synergy and muscular activation patterns (Dominici et al., 2011; Ivanenko et al., 2013).

In mammals, the spinal cord contains autonomously regulated networks that solely suffice to control alternating extensor-flexor activation of the limbs (Forssberg et al., 1980). There, repetitive rhythmic locomotor behavior may be evoked by local injections of neurochemical substances in spinally transected quadrupeds (Grillner and Zangger, 1979). This leads to locomotor primitives that are similar for spinal and healthy mammals (Desrochers et al., 2019). These networks closely resemble CPGs (McCrea and Rybak, 2008). While “[s]tudies of the neural control of animal locomotion certainly provide an important basis for such work on human walking … [one] should not expect that the results would inevitably be comparable. Decerebrate animals do not have the full gamut of descending inputs, and spinalized preparations have none.” (Capaday, 2002). The motor cortex has a privileged role in humans compared to other mammals as any interference of its downward projections results in locomotor deficits in humans. Our non-invasive assessment of cortico-synergy coherence allows for disentangling the organization of the locomotor primitives and for separating cortical and sub-cortical processes that possibly underlie the formation of muscle synergies. As we showed, it may serve to quantify cortical involvement in distinct primitives.

In humans, restoring walking capability after spinal lesions has been nearly impossible until recently (Wagner et al., 2018). In motor-complete spinal cord injury subjects, alternating locomotor-like movements together with rhythmic muscular activity can be evoked by stimulating the spinal cord at frequencies within the beta-frequency range (13–30 Hz) (Danner et al., 2015; Dimitrijevic et al., 1998). Arguably, the neural loops comprising beta-band synchrony are key components in the pattern-generating capacity within the spinal cord.

The two locomotor primitives associated with the motor cortex closely resemble those developing when toddlers start to walk independently (Dominici et al., 2011). To pinpoint these developments in more detail, longitudinal studies are needed. They may shed light on the differential neural organization of the muscle synergies. In view of our current cross-sectional assessments, we expect toddlers starting to walk independently to coincide with the emergence of the supplementary synergies and the accompanying cortico-synergy coupling.

### Limitations of the study

The present study found that the motor cortex predominantly interacts with two muscle synergies. Here, the strength of coupling between the motor cortex and these muscle synergies was assessed through cortico-synergy coherence. In neurophysiological sense, it is thought that such cortico-spinal coherence particularly reflects neural processes along the cortico-spinal tract operating at isolated frequencies. This means that neural processes cannot be captured in case the spectral interactions do not occur linearly. In that sense, it would be interesting to re-assess cortico-synergy coupling by examining cross-frequency measures to see if the functional coupling between the cortical areas and two muscle synergies with double support phase patterns persists.

## STAR★Methods

### Key resources table

**Table:** 

REAGENT or RESOURCE	SOURCE	IDENTIFIER
**Software and algorithms**		

MATLAB 2019a	MathWorks	www.mathworks.com/
FieldTrip	FieldTrip	www.fieldtriptoolbox.org/
SPM12	SPM12	www.fil.ion.ucl.ac.uk/spm/software/spm12/

### Resource availability

#### Lead contact

• Further information and requests for resources should be directed to and will be fulfilled by the lead contact, dr. Nadia Dominici (n.dominici@vu.nl).

#### Material availability

This study did not generate new unique reagents.

### Experimental model and subject details

#### Participants

All procedures were in full compliance with the Declaration of Helsinki for experiments involving humans. The local ethical committee of the Faculty of Behavioural and Movement Sciences Amsterdam (study protocols #VCWE-2016-082 and #VCWE-2020-006) approved the experimental procedures. Responsible researchers informed the adults and children’s parents about the study procedures and asked them to provide written informed consent prior to inclusion into the study. Participants visited the BabyGaitLab laboratory of the Department of Human Movement Sciences at the Vrije Universiteit Amsterdam. For the toddler measurements, at least one parent or legal guardian was present during the experiments along with the responsible researchers. Our laboratory settings and experimental procedures were adapted to children so that any risk was equal or lower to that of walking at home.

Eighteen typically developing toddlers and adults were included in the study. Population characteristics of both groups can be found in Tables 1 and 2. A local child healthcare clinic qualified the toddlers (gestational age: 276 ± 8 days; mean ± SD) as healthy: no neurological disorders, nor any significant health or developmental problems. Toddlers were selected based on their time since onset of unsupported walking and had 6.4 ± 1.0 months of independent walking experience when they visited the lab. We defined the first independent steps as the ability to perform four or more consecutive strides without the need for any physical support during walking and asked the parents to report this moment to us. We scheduled a recording session for each toddler around six months after the first independent steps to guarantee that all toddlers in the group had about the same walking experience. Adult subjects were recruited from a student pool and were awarded study credits for their educational curriculum. Exclusion criteria for the adult population were a self-reported history of neurological disorders.

**Table:** Characteristics of the typically developing (TD) toddlers and the number of analyzed strides

Toddlers						
Participant	Gender	Age (months)	Weight (kg)	Height (cm)	Walking onset (months)	# strides
TD1	F	18.2	10.5	83	11.9	457
TD2	M	17.4	10.5	80	11.7	133
TD3	M	20.8	14.0	96	15.3	468
TD4	F	23.1	13.2	91	16.7	570
TD5	F	19.4	11.7	84	14.8	349
TD6	M	18.6	12.2	81	12.9	389
TD7	F	19.8	11.2	82	13.0	648
TD8	F	20.1	11.0	81	14.0	313
TD9	M	23.6	12.2	88	14.2	126
TD10	F	19.3	11.9	81	13.0	94
TD11	M	16.5	11.3	79	10.6	431
TD12	F	17.5	10.7	82	11.3	389
TD13	M	23.0	13.5	95	15.7	779
TD14	F	19.3	12.5	81	13.1	752
TD15	F	19.0	10.4	82	12.4	824
TD16	M	20.9	11.4	84	15.0	687
TD17	M	19.1	12.9	78	12.9	306
TD18	F	19.8	10.4	84	13.1	288
Mean ± SD		19.7 ± 2.0	11.8 ± 1.1	84.0 ± 5.2	13.4 ± 1.6	447 ± 225

**Table:** Characteristics of the adults (A) and the number of analyzed strides

Adults					
Participant	Gender	Age (years)	Weight (kg)	Height (cm)	# strides
A1	F	21	59.9	172	416
A2	F	19	77.6	177	386
A3	F	21	65.7	173	382
A4	M	22	67.9	171	415
A5	F	22	58.5	175	363
A6	F	20	79.9	174	458
A7	M	19	66.7	187	396
A8	M	18	68.0	189	422
A9	F	19	57.0	170	434
A10	F	19	65.3	174	396
A11	F	20	66.1	165	387
A12	F	19	61.0	164	396
A13	F	46	57.0	172	403
A14	F	19	54.0	173	569
A15	F	19	54.4	165	383
A16	F	18	51.0	164	376
A17	F	26	47.0	154	457
A18	M	25	61.0	178	433
Mean ± SD		21.8 ± 6.4	62.1 ± 8.5	172.1 ± 8.2	415 ± 47

### Method details

#### Experimental procedures

When entering the laboratory, toddlers got time to familiarize with the environment and researchers before starting the experiment. The experimental design consisted of over-ground and treadmill walking. Over-ground trials were conducted at a lab space with the dimensions of ∼5.5 × 3.5 m, where participants naturally walked barefoot from one side of the lab to the other side at their preferred speed. Treadmill trials were performed at a pediatric treadmill, specifically designed for subjects of lower ages and suitable for adults (N-Mill 60 × 150 cm, Motek Medical B.V., Amsterdam, the Netherlands). Treadmill speed in toddlers was chosen to elicit either supported or unsupported walking. Toddlers were encouraged to walk independent of any type of physical support. Either holding one or two hand(s), stabilizing the trunk or some active body-weight support (i.e., physically supporting the body) was applied in case it helped to elicit continuous walking. Short walking trials (<2 min) were intermitted by rest breaks in between. Adults walked at a self-selected comfortable walking speed in 30 over-ground and six treadmill trials. Toddlers and adults performed a total of 447 ± 225 and 415 ± 47 strides, respectively (Tables 1 and 2).

#### Data acquisition

##### Kinematics

A Vicon motion capture system (Vicon Vero v2.2, Oxford, UK), with 10 cameras placed around the walking path, sampled bilateral kinematic and video (Vue Vicon camera) data at 100 Hz. Twenty-three infrared reflective markers (diameter: 14 mm) were attached to the participant’s body with adhesive tape. For the analyses of the current paper, we specifically focused on the markers attached to the bilateral lateral malleoli and fifth metatarso-phalangeal joint, and the bilateral heel marker in the adults’ group. EEG, EMG, kinematics, and video feed were simultaneously acquired and synchronized.

##### Electromyography

Bipolar muscular activity was recorded of 20–24 muscles simultaneously. The complete set of 24 muscles consisted of the bilateral activity of the tibialis anterior (TA), gastrocnemius lateralis (GL), gastrocnemius medialis (GM), soleus (SOL), rectus femoris (RF), vastus lateralis (VL), vastus medialis (VM), biceps femoris (BF), semitendinosus (SEM), tensor fasciae latae (TFL), gluteus maximus (GLM), and erector spinae (ES). In preparation, the participant’s skin was slightly rubbed with alcohol at the locations where the electrodes were attached. Ag/AgCl electrode pairs for toddlers and adults (Mini Golden electrodes, Cometa, 15-mm-diameter electrodes, acquisition area of 4 mm^2^; and Medico, Ambu® Blue Sensor®, respectively) were attached to the skin over the muscle belly according to standard recommendations for minimizing cross-talk between adjacent muscles (Hermens et al., 2000). In toddlers, movement artifacts were additionally minimized by fixating the electrodes and wireless EMG sensors to the thighs and shanks using elastic gauzes. EMG signals were recorded using two 16-channel wireless EMG systems (Wave Plus wireless EMG system with mini probes, Cometa, Milan, Italy). They were amplified with a gain of 1,000, and online band-pass filtered between 10 and 500 Hz before sampling at 1 kHz.

##### Electro-encephalography

Toddlers and adults wore a 32-channel and 64-channel EEG cap (WaveGuard^TM^, ANT-Neuro, Enschede, the Netherlands; and TMSi REFA, TMSi Twente, the Netherlands, respectively) to record electrical cortical activity. For toddlers, the EEG cap was pre-gelled before mounting it on the head. After placement, with the help of a blunt plastic little stick we distributed the impedance gel (SonoGel, Bad Camberg, Germany) homogeneously and, if required, we injected additional gel to improve the impedance between the skin and electrode. In adults, the EEG cap was aligned to the fiducials. We injected impedance gel in every channel to lower the impedance. Toddler and adult nylon EEG caps with sintered electrodes were mounted in accordance with the international 10–20 system. EEG data were sampled at a frequency of 2,048 Hz (eego^TM^ mylab, ANT B.V., Enschede, the Netherlands). Reference and ground electrodes were mounted on channel CPz and AFz, respectively. To compare the cortical activity of toddlers and adults, the 64-channel configuration of adults was reduced to match the 32 channels of toddlers.

### Quantification and statistical analysis

#### Data analysis

##### Kinematics and gait events

Foot-contact and foot-off events of adult participants were detected using a peak-detection algorithm based on the bilateral heel marker and its first-order derivative (Roerdink et al., 2008). In toddlers, gait events were determined from digital video recordings and marker trajectories using Vicon Nexus software (Vicon, Oxford, UK). Gait events were independently assessed by at least two experienced researchers and confirmed with kinematic data. Events associated with gait initiation/termination and turning as well as non-locomotor movements (e.g., jumps) were discarded from further analysis. Here, we defined a complete stride (i.e., a gait cycle) from the moment the right foot first touched down until the next consecutive touch-down of the right foot.

##### Pre-processing and artifact removal electrophysiological data

EMG data were visually inspected to identify artifacts and to remove the corrupted data segments from further analysis. EMG time series were high-pass filtered at 30 Hz (bidirectional fourth-order Butterworth filter), and notch-filtered (bidirectional fourth-order Butterworth filter around *k*·50 Hz; *k* = 1, 2, 3, 4). EMG signals were full-wave rectified by taking the modulus of the analytic signal (Boonstra and Breakspear, 2012). Rectified EMG ($\overset{˜}{\text{EMG}}$) was then low-pass filtered at 5 Hz (bidirectional fourth-order Butterworth filter).

Sensor-level EEG was pre-processed using custom scripts in MATLAB (ver. 2019a, MathWorks, Natick, USA) and the FieldTrip toolbox (https://www.fieldtriptoolbox.org/) (Oostenveld et al., 2011). Raw data were tested for outlier samples and interpolated via cubic splines. To test for outlier samples, values exceeding ten times the SD of a channel after high-pass filtering were identified and subsequently corrected by cubic spline interpolation of the non-filtered data. Next, EEG time series were demeaned, bandpass- (bidirectional second-order Butterworth filter between 1 and 200 Hz) and notch-filtered (bidirectional first-order Butterworth filter around *k*∗50 Hz; *k* = 1, 2, 3, 4). Channels were checked for flat lines (smaller than 2^−52^ μV) and excessive amplitudes. For the latter we used the following criteria: (1) a channel-specific SD of three times higher than the mean SD across channels, (2) a channel-specific maximal value of ten times higher than the mean or median maximal value of all channels and (3) a SD of 100 times smaller than the mean SD across channels. Artifact-sensitive channels were excluded, and their activity was spatially reconstructed using spherically spline interpolation based on available neighboring channels. Single trials were discarded from further analysis when more than 10 channels were marked as artifact sensitive. This led to the exclusion of 1.2% of the trials. An average of 1.5 ± 2.2 channels were considered as bad across all trials of all participants. After ‘bad’ channel detection, EEG time series were re-referenced to an average common reference. Data of every trial were subjected to an independent component analysis (ICA) using a fastICA-algorithm with online bias detection and random seed generator. The convergence parameter of the maximal number of iterations was set to 5,000. Criteria to consider modes as artifacts were a median frequency lower than 1 or higher than 100 Hz (movement and muscular artifacts, respectively). Mixing matrix components dominated by frontal channels (eye movements) were considered as eye-movement artifacts. Sensor-level EEG was reconstructed after discarding the artifact-sensitive modes from the mixing matrix. For our study population, 3.4 ± 4.0 components were discarded across all trials of all participants.

##### Muscle synergies

Envelope EMG time series were time- and amplitude-normalized by resampling the epochs from two successive foot touch-down events of the right leg to 201 samples (Dominici et al., 2011) and normalizing the amplitude to their Euclidian norm (Banks et al., 2017), respectively. Minima were subtracted from the normalized patterns (Figure S1). Non-negative matrix factorization (NNMF) decomposed the group average muscular envelopes (i.e., the average of all strides within the group) into muscle synergies. Generally, NNMF factorizes multivariate EMG (dimensions: muscles × time samples) into a low-dimensional set of non-negative modes, or more formally:

(Equation 1) $$\text{EMG}\approx \sum_{\text{i}=1}^{\text{N}=4}{\text{P}}_{\text{i}}\cdot {\text{W}}_{\text{i}}+\text{є}$$

where the product of the temporal activation patterns (*P*; time samples × synergies) and their muscle weighting coefficients (*W*; synergies × muscles) of *N* synergies approximate *EMG*, the EMG envelopes; є denotes the residual error. Factorization parameters were set to 100 repetitions and 1,000 iterations. Termination parameters for residual size and relative change in *P* and *W* were set to 10^−6^. NNMF-output was estimated based on multiplicative update and alternating least-squares algorithms (Berry et al., 2007), where *P* and *W* of the former factorization served as starting values for the latter factorization. To determine the reconstruction accuracy of the extracted synergies, we computed the cumulative variance. In both groups, four synergies were extracted to reproduce the EMG envelopes (Figures 2A, 2B, and S1), accounting for 98.7% of variance of the mean patterns of toddlers and 91.5% of variance in adults.

Given a signal *EMG*, the NNMF can be computed by minimizing the Frobenius norm ${\left\|\text{EMG}-\left(\text{P}\cdot \text{W}\right)\right\|}_{\text{Fro}}$, which yields a set of weights W, with which one defines the virtual activation patterns ${\text{P}}_{\text{vir}}$ as.

(Equation 2) $${\text{P}}_{\text{vir}}=\overset{˜}{\text{EMG}}\cdot {\text{W}}^{-1}$$

Here ${\text{W}}^{-1}$ denotes the Moore-Penrose inverse of *W* and $\overset{˜}{\text{EMG}}$ the EMG time series before low-pass filtering – note that we used the dot notation to indicate the mere matrix multiplication. The virtual activation patterns adequately represent the waveshapes of the basic activation patterns *P* including high-frequency components. Virtual activation patterns were included in the coherence analyses with the EEG (Zandvoort et al., 2019, 2021).

We would like to note that in contrast to the original signals *EMG* the virtual activation patterns ${\text{P}}_{\text{vir}}$ should not be considered rectified electromyograms, since for |є|>0 the rank-reduction in (1) may yield ${\text{P}}_{\text{vir}}>0$. We did encounter negative samples (see Table S2) rendering this note important. By the same token, however, we can stress that ${\text{P}}_{\text{vir}}$ closely resembled *P*; correlating ${\text{P}}_{\text{vir}}$ and *P* revealed correlation coefficients between 0.95 and 0.99.

##### Source modeling models

To localize cortical sources that exhibit coherence with the muscle synergies (i.e., virtual activation patterns), we applied a source modeling approach called beamforming. Using such algorithms, the inverse problem (i.e., not knowing where brain activity originates from) can be solved. First, we created a forward model comprising a volume conduction, source, and electrode model. Realistic five-layer head models were constructed using age-specific structural MRIs from the Neurodevelopmental MRI Database (Richards et al., 2016; Sanchez et al., 2012). We selected averaged MRIs from the database that were close to the average age of the toddler and adult population, which were 18 months and 20–24 years, respectively (Richards et al., 2016; Sanchez et al., 2012). White matter, gray matter, and cerebral spinal fluid segments were created from probabilistic tissue maps. Scalp and skull segments were modeled at the outer voxels of the inner-skull tissue maps. The five segmented tissues were meshed into hexahedrons. A realistically shaped five-shell volume conduction model was constructed from the meshed tissue volumes using the SIMBIO-software. Conductivity values of the isotropic compartments were set to 0.43 (scalp), 0.01 (skull), 1.79 (cerebral spinal fluid), 0.33 (gray), and 0.14 (white) Siemens/meter (O'Reilly et al., 2021). The gray segment of the volume conduction model was discretized into a three-dimensional grid of 5 mm^3^ (i.e., source model). We used an inward shift of 15 mm from the scalp. An age-matched template electrode model was (non-)linearly warped onto the outer surface of the head model so that the distances between electrodes and scalp were minimized. Forward solutions by means of lead fields were estimated using the volume conduction model, source model and three-dimensional sensor positions. Lead-field matrices uniquely solve the forward problem and describe the propagation of the electrical activity from cortical dipoles to the sensors in three orthogonal directions.

##### Cortical source localization and reconstruction

Dynamic Imaging of Coherent Sources (DICS) beamformers were estimated to localize the cortico-synergy coherence between the EEG channels and muscle synergies (Groβ et al., 2001). Spatial filters of DICS-beamformers were optimized by maximizing the coherence between the sensor-level EEG and virtual activation patterns. That is, the cross-spectral density matrices of the EEG were extended by employing the virtual activation patterns as reference channel. Generally, coherence describes phase locking at iso-frequencies between two signals while being weighted by the signals’ amplitudes and is defined as:

(Equation 3) $${\text{C}}_{\text{xy}}\left(\omega \right)=\frac{{\left|\left\langle \left|{\text{S}}_{\text{X}}\left(\omega \right){\text{S}}_{\text{Y}}\left(\omega \right)\right|{\text{e}}^{\text{i}\left(\varphi_{\text{x}}\left(\text{ω}\right)-\varphi_{\text{y}}\left(\text{ω}\right)\right)}\right\rangle \right|}^{2}}{\left\langle {\left|{\text{S}}_{\text{X}}\left(\omega \right)\right|}^{2}\right\rangle \left\langle {\left|{\text{S}}_{\text{Y}}\left(\omega \right)\right|}^{2}\right\rangle }$$

where ${\text{C}}_{\text{xy}}\left(\omega \right)$ denotes the squared coherence as a function of frequency, ⟨⋅⟩ the expectation value, and *S* the Fourier transform of the signals *x* and *y*.

We specifically targeted the beta-frequency band (13–30 Hz) as coherence comprising sensor-level EEG yields highest coherence magnitudes at this frequency range. To estimate coherence beamformers, we first resampled the virtual activation patterns to 2,048 Hz so that it matched the EEG’s sampling rate. Both EEG data and virtual activation patterns were down sampled to 256 Hz for computational reasons. Resampled time series of sensor-level EEG and virtual activation patterns were spectrally transformed using short-time Fourier transforms and Slepian sequences of 200ms. The frequency of 21.5 Hz was selected as center frequency and spectral smoothing was set to 8.5 Hz. These temporal and spectral parameters resulted in Slepian sequences with two tapers. From the Fourier transformed data, we estimated the cross- and auto-spectra for all strides available. Cross- and auto-spectra were averaged over the time and frequency dimension for every stride. From all strides available for every subject, we took the median spectra to obtain subject-specific estimates. Beamformers were constrained to be real valued to avoid phase shifts between channels. Phase shifts would violate the assumption of linear transformations between sensors and sources (Westner et al., 2022). The orientation of dipoles did not have to be fixed so that moments were obtained in the three orthogonal directions. Note that after the application of principal component analysis during source reconstruction only the strongest moment was retained. The regularization parameter of the cross-spectral density matrix was set to 5%. We chose this value as a trade-off between the signal-to-noise ratio and spatial precision (Woolrich et al., 2011). Adding (a percentage of) mean sensor power to the diagonal of the cross-spectrum matrix will effectively broaden the passband and, hence, increases the filter’s signal-to-noise ratio (Brookes et al., 2008); by the same token, however, regularization will result in blurring of the output volumes.

Cluster-based permutation tests were employed to assess the coherence-based beamformers. The advantage of cluster-based permutation testing is that it does not require any assumptions about probability density functions (Maris and Oostenveld, 2007). Rather, their probability density functions are estimated by permuting the representations for several partitions by Monte Carlo estimations. Using permutation testing, one can also reduce the number of multiple comparisons. Permutation testing accomplishes this by thresholding based on clusters from neighboring samples rather than individual samples. This involves grouping test statistics of adjacent samples that exceed a certain predefined α-level. Clusters that exceed the α-level are considered significant (Nichols and Holmes, 2002). For the within-subject statistics, we iteratively performed permutation testing for each synergy (synergies 1–4) and group (toddlers and adults). We considered the subject-specific beamformer volumes that were computed over the entire gait cycle. We performed a one-sample t-test on these volumes for the contrast ‘coherence minus subject-specific mean coherence >0’. To do so, we first normalized individual coherence volumes by subtracting the mean coherence over all voxels from the subject-specific volumes. We note that the mean subtraction is equivalent to contrasting the coherence volumes without mean subtraction against their own mean. This entails that our statistical volumes do not reflect the absolute coherence strength, and that significant clusters are indicative of higher spatial coherence relative to all other voxels. After mean subtraction, coherence values are bounded between −1 and 1. To stabilize its variance, we applied a Fisher transform via the inverse hyperbolic tangents (i.e., *atanh(coh)*) function. In permutation testing, clusters were considered significant based on the intensity and size of the clusters. This can be realized via the weighted cluster mass (Hayasaka and Nichols, 2004; Maris and Oostenveld, 2007). The weighted cluster mass is sensitive to both amplitude of the test statistic and number of grid points. Permutation testing was performed with 4,096 randomizations and (cluster-) alpha of 0.005. The source localization volumes, as presented in Figure 2, are the result of power and cross-spectral averages over the entire gait cycle (from right-to-right foot contact).

##### Effect of side gait cycle events on cortical coherence sources

To ensure that the cortical grouping of the muscle synergies is not simply the result of our time-locking procedure (Figures S2 and S3), we again performed the source analysis by time locking from left-to-left foot contact. The cortical coherence sources defined from left-to-left foot contact were similar to those obtained with time locking from right-to-right foot contact (Figures S4–S7). That is, synergies 1 and 3 resembled significant beta-band coherence with the motor cortex whereas synergies 2 and 4 did not. Again, the observed coherence was found in both toddlers and adults.

Moreover, the coherence sources turned out to be lateralized for some of the synergies. The left hemisphere seems to be more dominantly involved as it resembled highest beta-band coherence for synergies 1 and 3 (Table S1). This lateralization was evident in toddlers and adults and was independent of the time-lock event (Figures S2–S7).

##### Development changes of the cortical representations

To test for between-group, developmental differences, we compared the toddlers’ volumes against adults. The beamformer volumes of toddlers and adults did have different geometry, hampering valid statistical comparisons between groups. Hence, we first spatially normalized the three-dimensional coherence images (Ashburner and Friston, 2005; Friston et al., 1994). To estimate the optimal normalization parameters, we set the bias regularization and full-width half maximum (FWHM) to 0.0001 (very light) and 60 mm, the regularization parameters to [0 0.001 0.5 0.05 0.2], the fudge factor smoothing to 5 mm and the sampling distance for the estimation of modeling parameters to 2. Warped volumes were interpolated using a nearest neighboring approach. Additionally, the warped toddler and original adult volumes were smoothened with an FWHM of 1 voxel. Like the within-group statistics, source data were mean-subtracted, and $\tilde{\text{c}}=\text{atanh}\left(\text{coh}\right)$. Resulting volumes were subjected to two-tailed independent t-tests using permutation testing to statistically assess the toddler-adult contrast (Figure 2C).

In line with the within-subject beamformer findings of the main text, significance was only found for synergies 1 and 3 in the between-group statistics (Figures S8–S10). For these synergies, mean coherence differences were both positive and negative for toddlers and adults. Positive significant clusters (toddlers > adults) were mainly found in the postcentral and parietal gyri. Negative significant clusters (toddlers < adults) were localized more frontal. Note that the locations of both cluster types are in line with the within-subject statistics.

##### Source reconstruction

To obtain reconstructed activity at the cortex, sensor-level EEG data were filtered to reconstruct the time series at the regions of interests (ROIs). ROIs were selected from the LONI Probabilistic Brain Atlas (LPBA-40) (Fillmore et al., 2015). Here, we selected the linear filters at a 5-mm circle around the voxel that exhibited maximal coherence as found within the precentral and postcentral gyri. After source reconstruction, we only kept the source time series comprising the maximal dipole moment. We took the first principal component from the three orthogonal dipole directions. The time series comprising the maximal dipole moment were considered for the estimation of the time-dependent cortico-synergy coherence.

##### Time-frequency cortico-synergy coherence

Auto- and cross-spectra as a function of time and frequency were obtained from source-reconstructed EEG and virtual activation patterns of entire walking trials. Auto- and cross-spectra were obtained with short-time Fourier transforms with Hanning windows of 200ms. These spectra were epoched from right-to-right foot contact and time-warped from 0 to 100% of the gait cycle. Spectra were averaged across all strides available for every subject. We computed coherence sample estimates according to Equation 3*.*

Both toddlers and adults exhibit modulation of beta-band coherence between motor cortex and synergies 1 and 3 throughout the gait cycle. Coherence is maximal around double support phases (Figure 3). To test for developmental changes, we then compared the beta-band coherence of toddlers and adults. To evaluate coherence changes as a function of time and frequency between toddlers and adults, we performed permutation testing on the time-frequency-coherence representations. We employed an independent t-test to evaluate the mean coherences of toddlers and adults. Number of randomizations was set to 8,196 with an alpha-level of 0.01 and a cluster-alpha of 0.01. Again, significance of clusters was assessed by the weighted cluster mass (Hayasaka and Nichols, 2004; Maris and Oostenveld, 2007).

We further studied the time-frequency coherence involving the left and right primary sensory cortices. For primary sensory cortex, the coherence patterns with synergies 1 and 3 modulated similarly to the coherences with the primary motor cortex (Figures S11 and S12). The sensory cortex yielded more pronounced clusters when comparing toddlers and adults than the motor cortex (lower rows of Figures S11 and S12). These toddler-adult differences between the motor and sensory cortex agrees with the muscle synergy representations at the cortex as presented in the main text (Figure 2). The cortical representations in adults were localized more frontally than the ones in toddlers. While adult sources were limited to the primary and supplementary areas of the motor cortex, the primary sensory cortex was also significant in toddlers for synergies 1 and 3. Also, to exclude effects on hemisphere selection we contrasted the time-frequency representations of left and right hemisphere from the primary motor and sensory areas in toddlers and adults. Findings revealed that there are no significant clusters when contrasting these time-frequency coherence representations of the right and left hemisphere.

To show that the observed coherence patterns were not a mere by-product of gait-cycle dependent power modulations, we estimated the event-related amplitude modulation in primary sensorimotor cortex and synergies 1 to 4 (Figure S13). The amplitudes of the cortical and synergy activity modulated as a function of the gait cycle. The cortical activity showed event-related beta-band (de-)synchronization locked to double and single support phases. Likewise, the beta-band amplitude of the virtual activation patterns was temporally aligned to the temporal patterns of the synergies. For the coherence patterns involving synergies 1 and 3 this means that the maximal coherence as observed during the double support phases did not emerge due to low power from the primary motor cortex and/or synergies.

## Data Availability

• Data will be shared by the lead contact upon request. • Code will be shared by the lead contact upon request. • Any additional information required to reanalyze the data reported in this paper is available from the lead contact upon request.
