# Supplementary material for: Cortical contributions to locomotor primitives in toddlers and adults
Source: iScience. 2022 Sep 28;25(10):105229. doi: 10.1016/j.isci.2022.105229 (PMC9576581; doi:10.1016/j.isci.2022.105229)
Supplement: Document S1. Figures S1–S14 and Tables S1 and S2 [file mmc1.pdf]

iScience, Volume *25*

Supplemental information

Cortical contributions to locomotor primitives in toddlers and adults

Coen S. Zandvoort, Andreas Daffertshofer, and Nadia Dominici

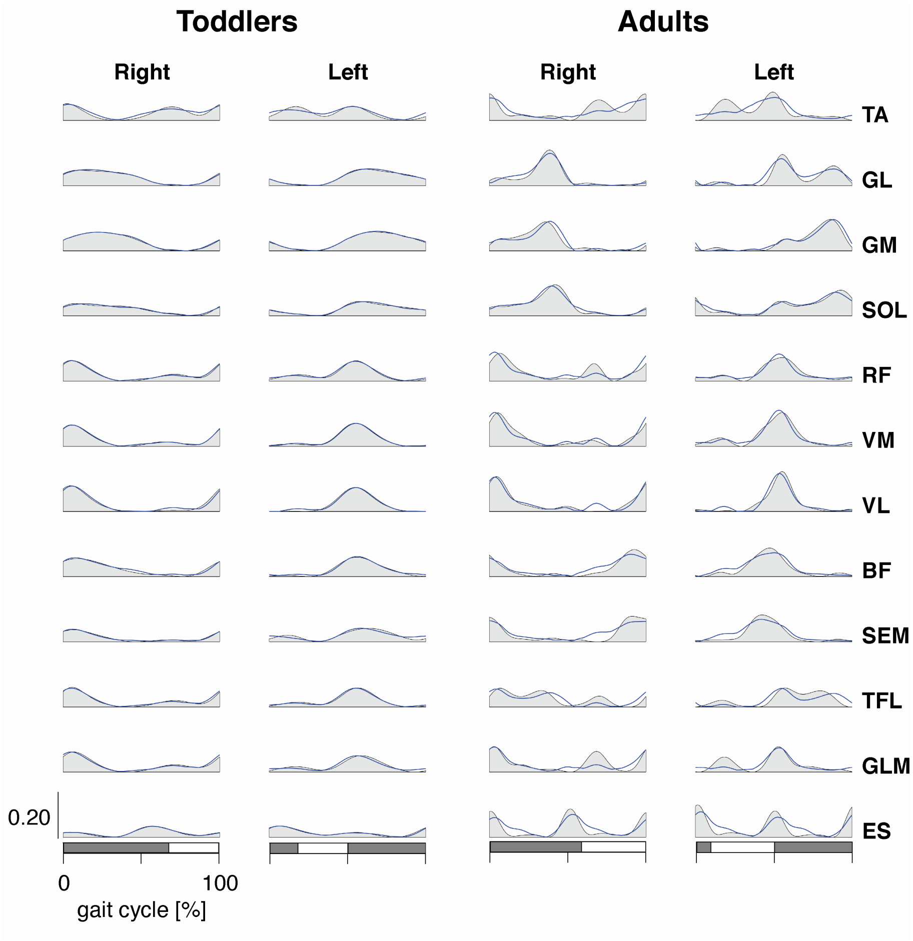

Figure S1. Bilateral muscular activation patterns of toddlers and adults, related to Figure 2. Patterns represent ensemble averages for both groups and are amplitude-normalized (i.e., EMG envelopes divided by their norm over time) and time-locked from right-to-right foot contact (percentage of the gait cycle). Thin blue lines depict reconstructed EMG-activity based on the inner product of the basic activation patterns and muscle weightings. TA, tibialis anterior; GL, gastrocnemius lateralis; GM, gastrocnemius medialis; SOL, soleus; RF, rectus femoris; VM, vastus medialis; VL, vastus lateralis; BF, biceps femoris; SEM, semitendinosus; TFL, tensor fasciae latae; GLM, gluteus maximus; ES, erector spinae. Horizontal bars below represent average stance (grey) and swing phases (white).

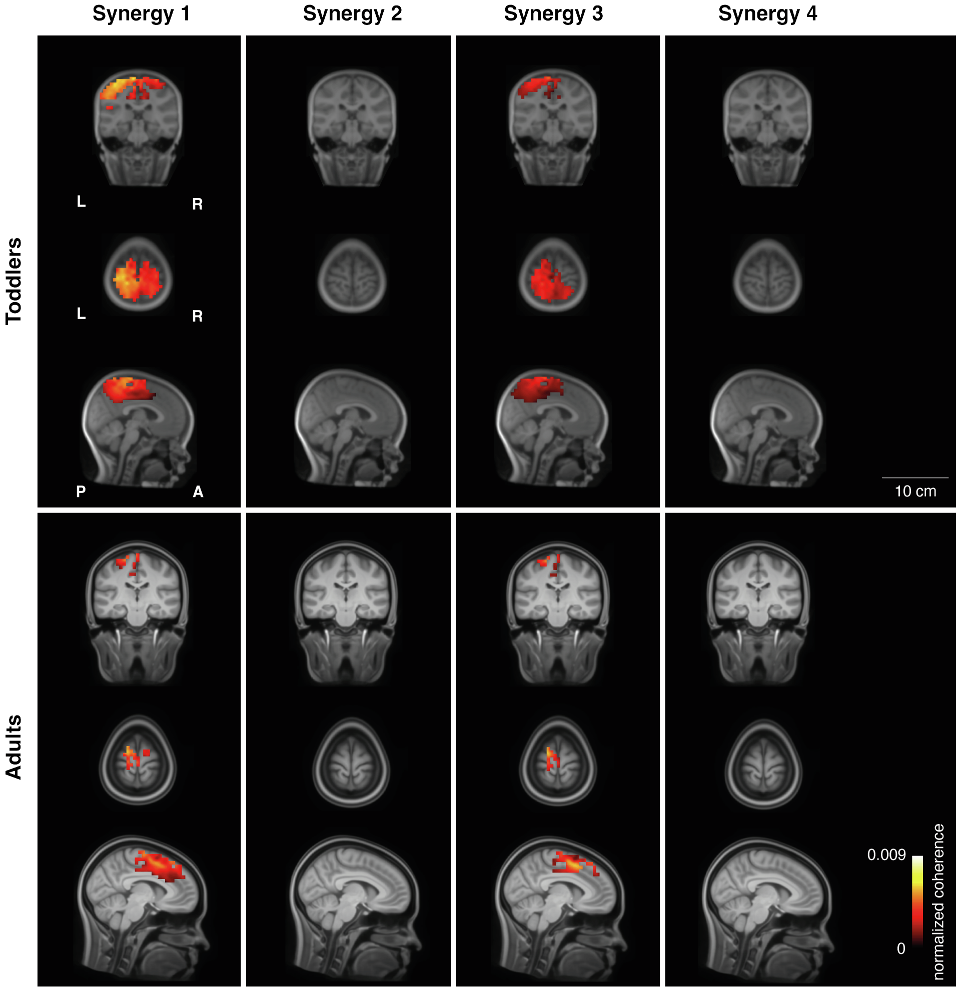

Figure S2. Cortical beta-band coherence representations projected on age-matched MRIs for toddlers (top) and adults (bottom) over the gait cycle computed from right-to-right foot contact, related to Figure 2. Coherence sources are like those presented in Figure 2 of the main text. The colored dimension represents the coherence voxels that significantly exceeded the subject's mean coherence. Colored sources are masked by the voxels that turned out significant from one-sided t-testing ($p < 0.005$; Figure S3). L: left; R: right; P: posterior; A: anterior.

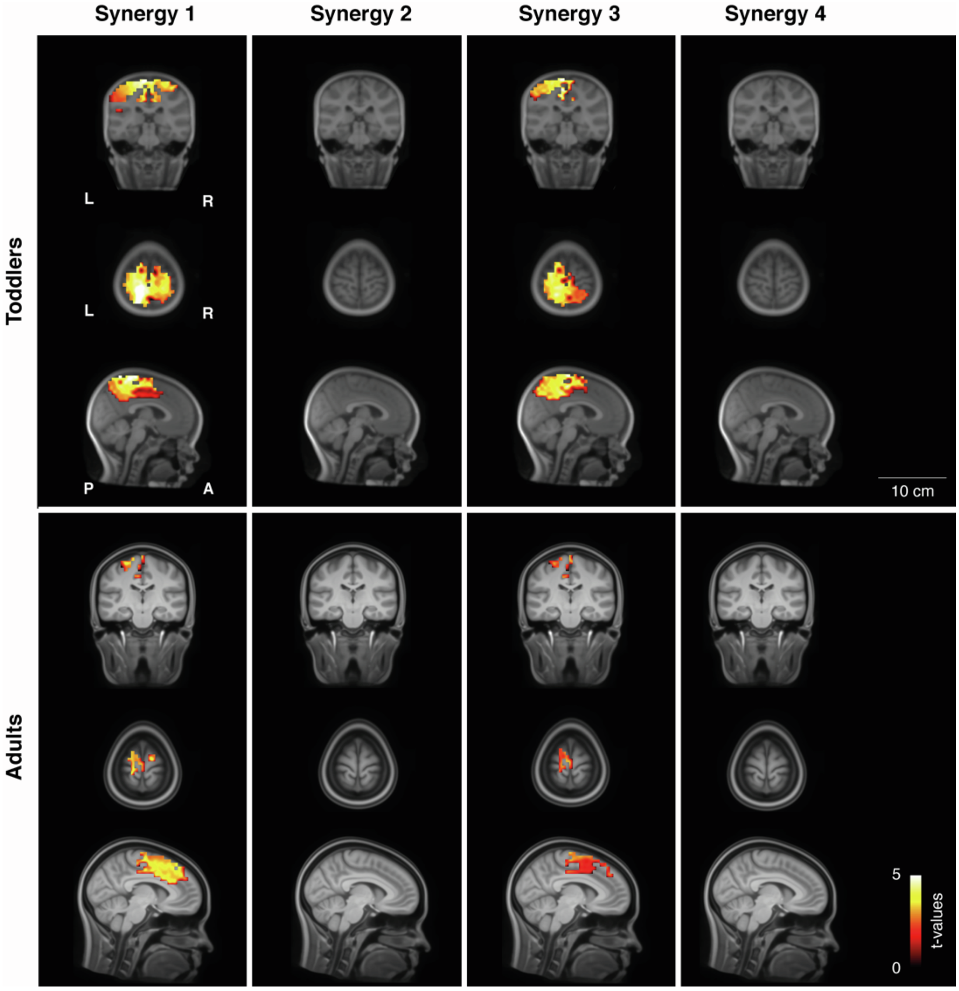

Figure S3. Statistical representations for toddlers (top) and adults (bottom) over the gait cycle computed from right-to-right foot contact, related to Figure 2. Cortical beta-band volumes were subjected to one-sided t-tests for the contrast 'coherence minus subject-specific mean coherence > 0' ($p < 0.005$). Significant sources for these contrasts are projected onto age-matched template MRIs. L: left; R: right; P: posterior; A: anterior.

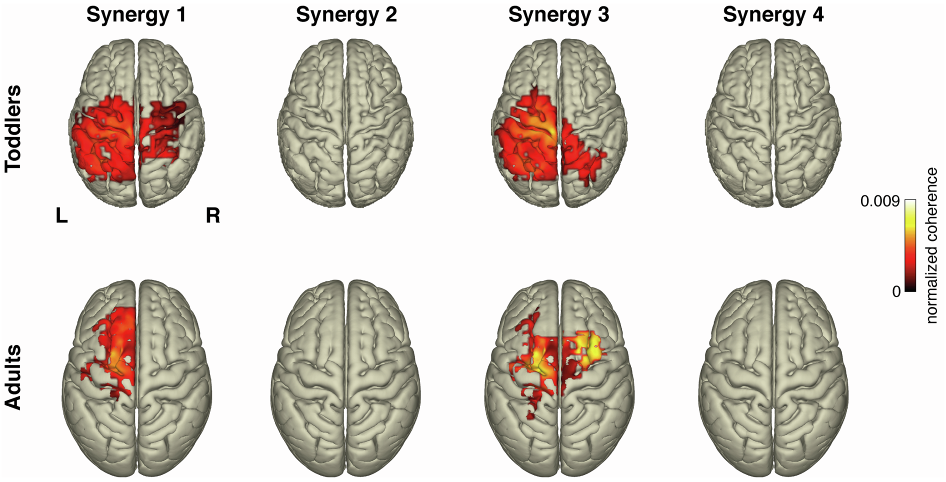

Figure S4. Beta-band coherence representations for toddlers (top) and adults (bottom) when source reconstruction is applied from left-to-left foot contact, related to Figure 2. Cortical volumes are masked by the voxels that were significant when testing the statistical contrast 'coherence minus subject-specific mean coherence > 0' ($p < 0.005$; Figure S6). L: left; R: right.

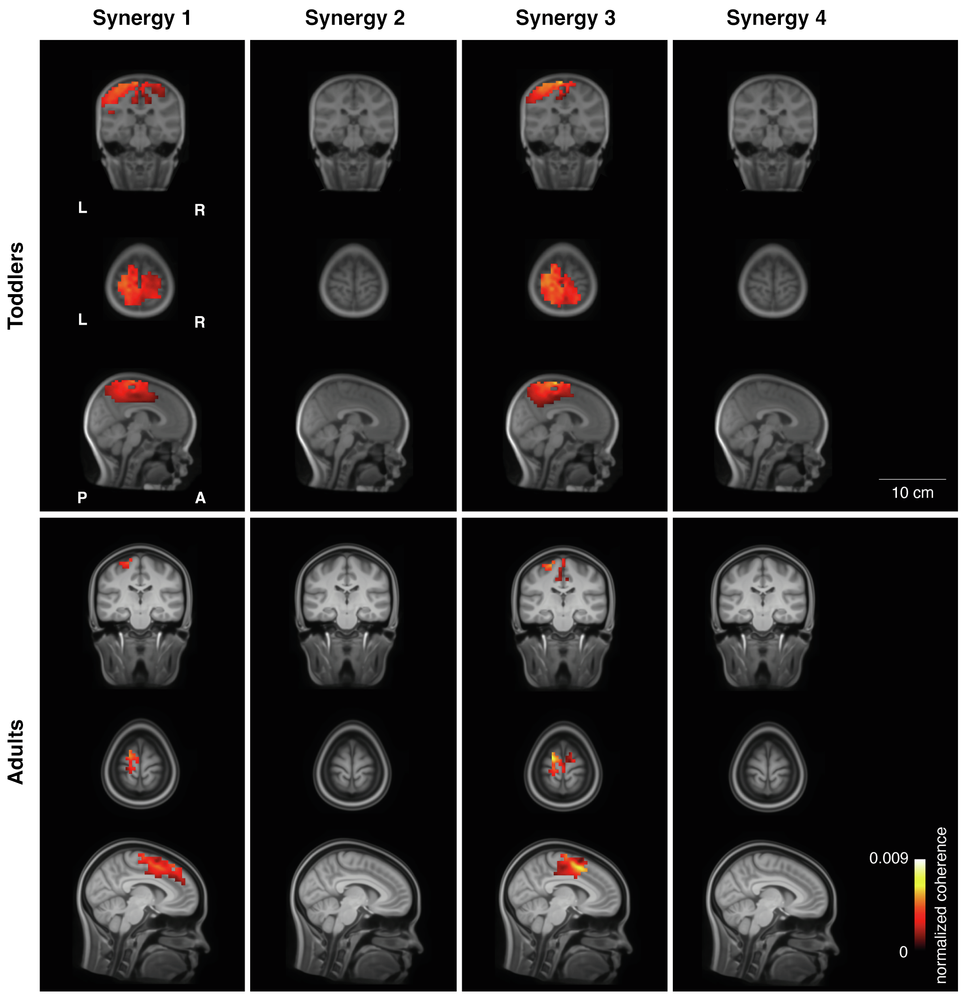

Figure S5. Beta-band coherence representations for toddlers (top) and adults (bottom) when source reconstruction is applied from left-to-left foot contact, related to Figure 2. Sources are identical to those presented in Figure S4. Cortical volumes are masked by the voxels that were significant when testing the contrast 'coherence minus subject-specific mean coherence > 0' ($p < 0.005$; Figure S6). L: left; R: right; P: posterior; A: anterior.

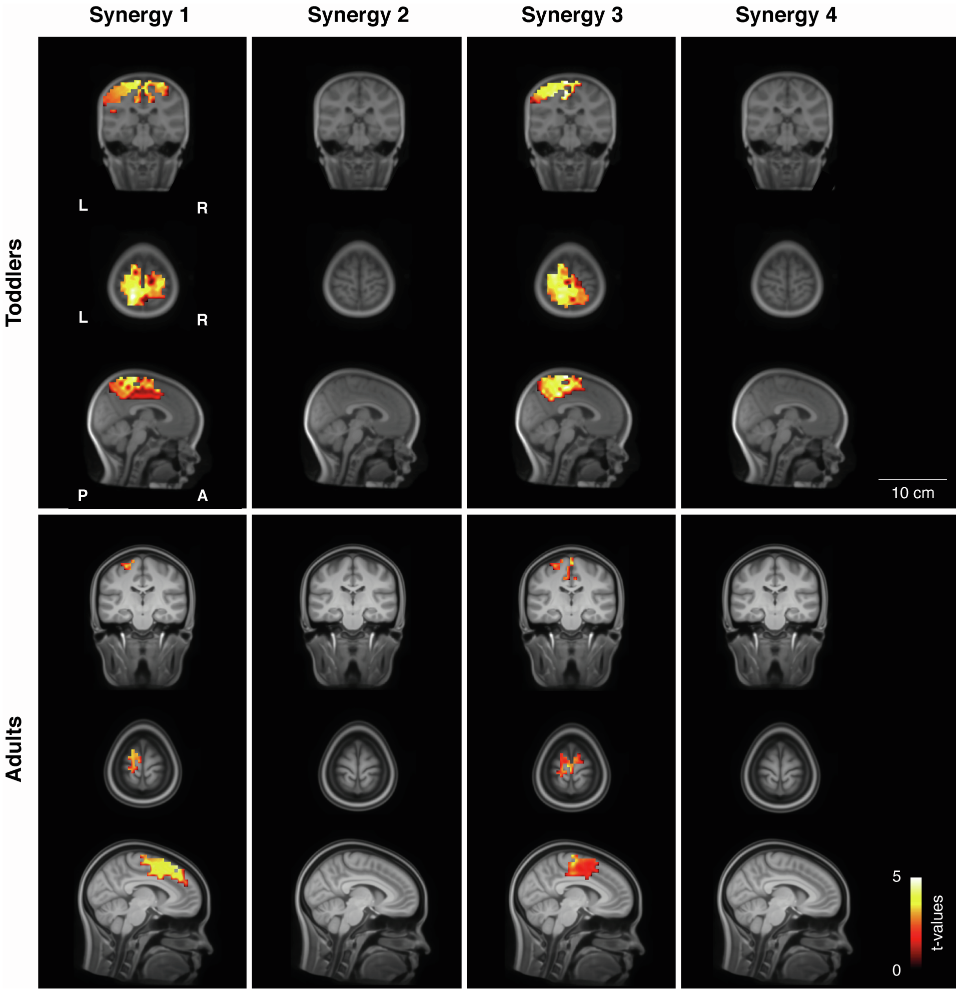

Figure S6. Statistical representations for toddlers (top) and adults (bottom) when time locking from left-to-left foot contact, related to Figure 2. Cortical beta-band volumes were subjected to one-sided t-tests for the contrast 'coherence minus subject-specific mean coherence > 0' ($p < 0.005$). Significant sources for these contrasts are projected onto age-matched template MRIs. L: left; R: right; P: posterior; A: anterior.

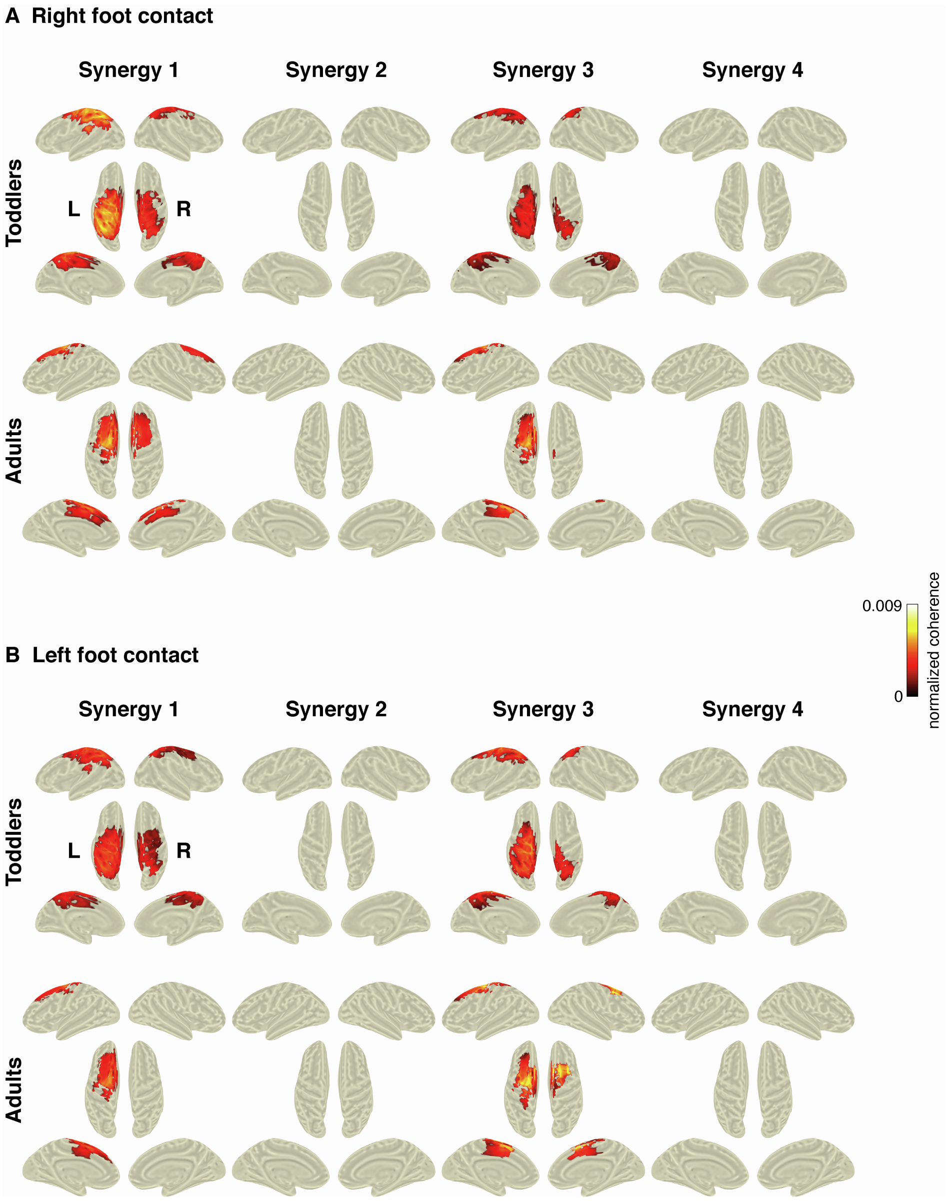

Figure S7. Beta-band coherence projected on inflated surfaces for toddlers (top) and adults (bottom), related to Figure 2. (A) Right and **(B)** left foot contact correspond to the event used for time locking during source analysis. Cortical volumes were subjected to one-sided t-tests for the contrast 'coherence minus subject-specific mean coherence > 0' ($p < 0.005$) to create a mask to identify cortical areas resembling significant coherence. L: left; R: right.

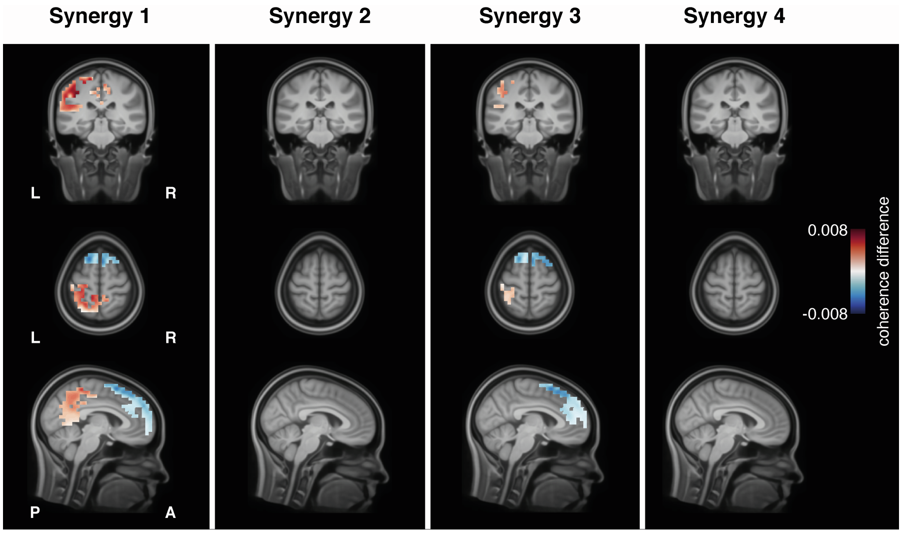

Figure S8. Toddler-adult differences of the cortical muscle synergy representations presented as mean coherences, related to Figure 2. Source-localized coherence volumes of toddlers and adults were subjected to statistical tests to evaluate for between-group differences. Sources have been defined as mean coherence difference between toddlers and adults for the contrast 'Toddlers minus Adults'. Hence, a positive coherence difference indicates a higher coherence for toddlers compared to adults. Cortical volumes are masked by the voxels that were significant when testing the contrast ($p < 0.025$; Figure S9). L: left; R: right; P: posterior; A: anterior.

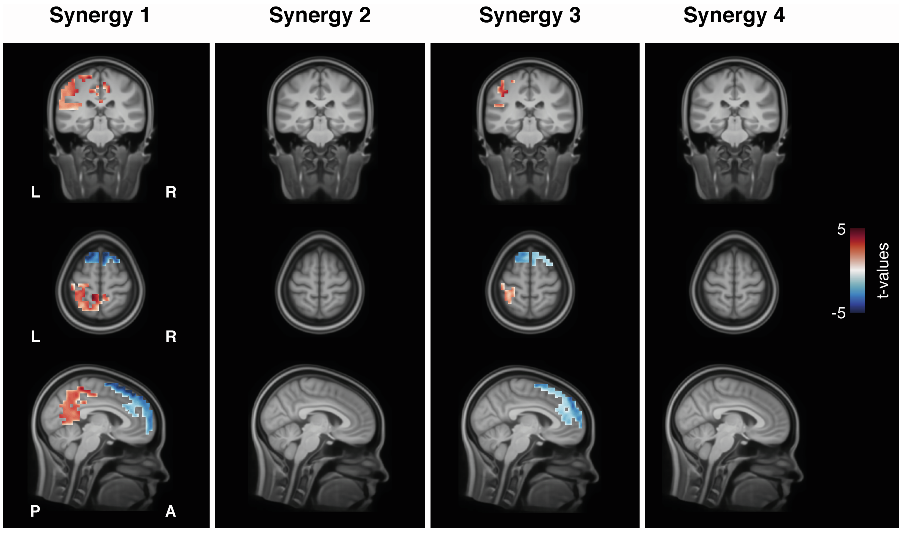

Figure S9. Toddler-adult differences of the cortical muscle synergy representations presented as significant t-values after unpaired t-testing between the two groups, related to Figure 2. Source-localized coherence volumes of toddlers and adults were subjected to statistical tests to evaluate for between-group differences. Sources have been based on the contrast 'Toddlers minus Adults'. Hence, positive t-values indicate higher coherence for toddlers compared to adults. Cortical volumes are masked by the voxels that were significant when testing the contrast ($p < 0.025$). L: left; R: right; P: posterior; A: anterior.

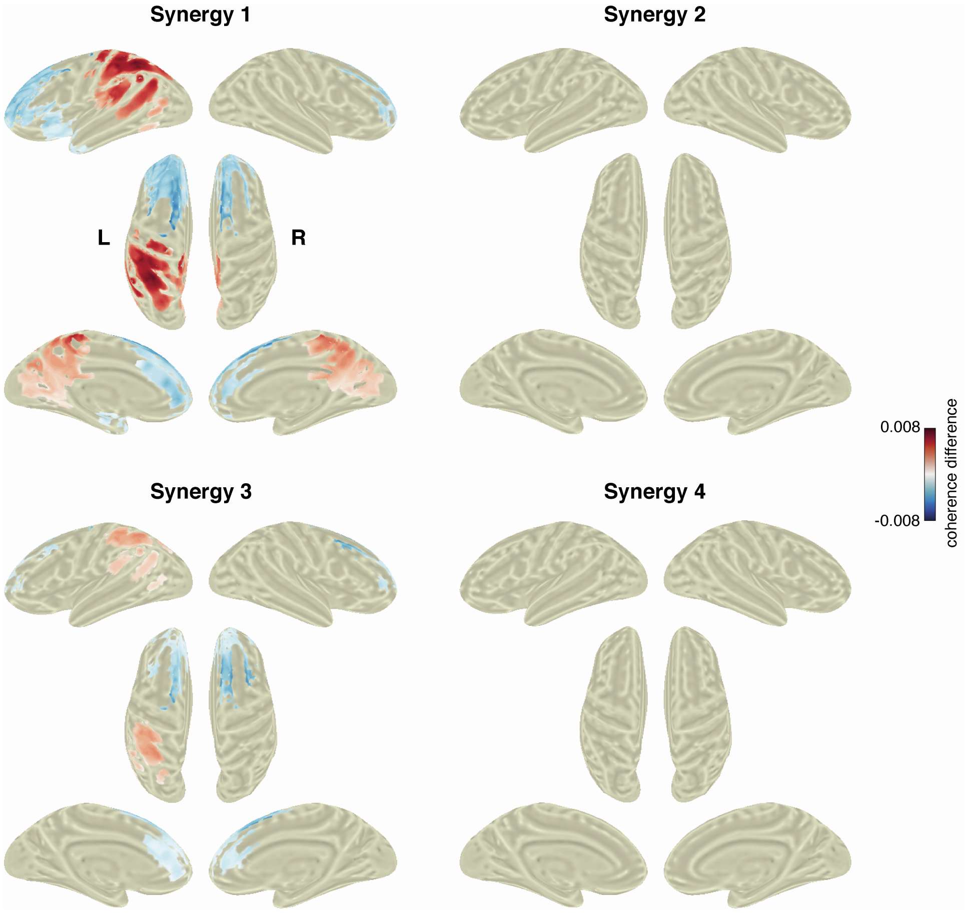

Figure S10. Between-group statistics of the beta-band coherence projected on inflated surfaces, related to Figure 2. Sources have been based on the contrast 'Toddlers minus Adults'. Hence, positive coherence differences indicate higher coherence for toddlers compared to adults. Cortical volumes are masked by the voxels that were significant when testing the contrast ($p < 0.025$). L: left; R: right.

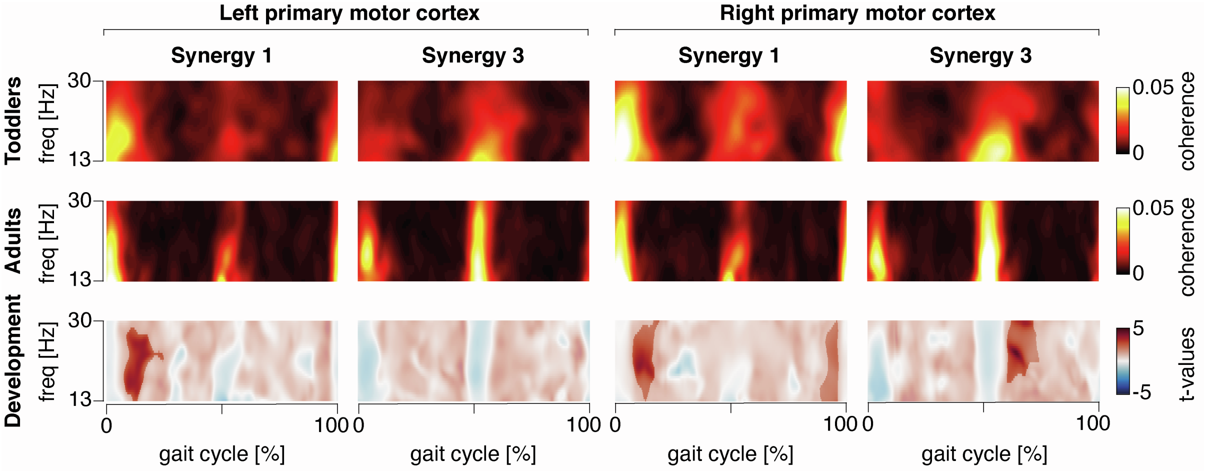

Figure S11. Time-frequency magnitude-squared coherence between bilateral primary motor cortices and synergies 1 and 3, related to Figure 3. Gait cycle is defined from right-to-right foot contact. Time-frequency coherences are pooled over participants for every synergy pattern for toddlers (upper row) and adults (middle row). Opaque colors indicate significant t-values for the developmental changes (lower row). Positive t-values yield higher coherence for toddlers compared to adults. freq: frequency

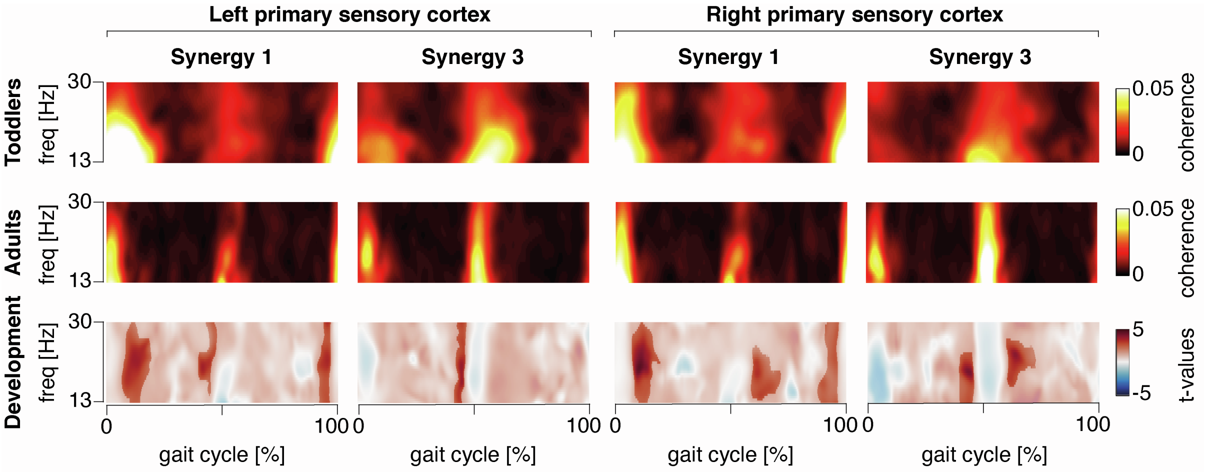

Figure S12. Time-frequency coherence between primary sensory cortices and synergies 1 and 3, related to Figure 3. Similar format as Figure S11. Developmental coherence changes are larger in the primary sensory cortices compared to the primary motor cortices (lower row of Figure S11). Opaque colors indicate significant t-values for the developmental changes (lower row). Positive t-values yield higher coherence for toddlers compared to adults. freq: frequency

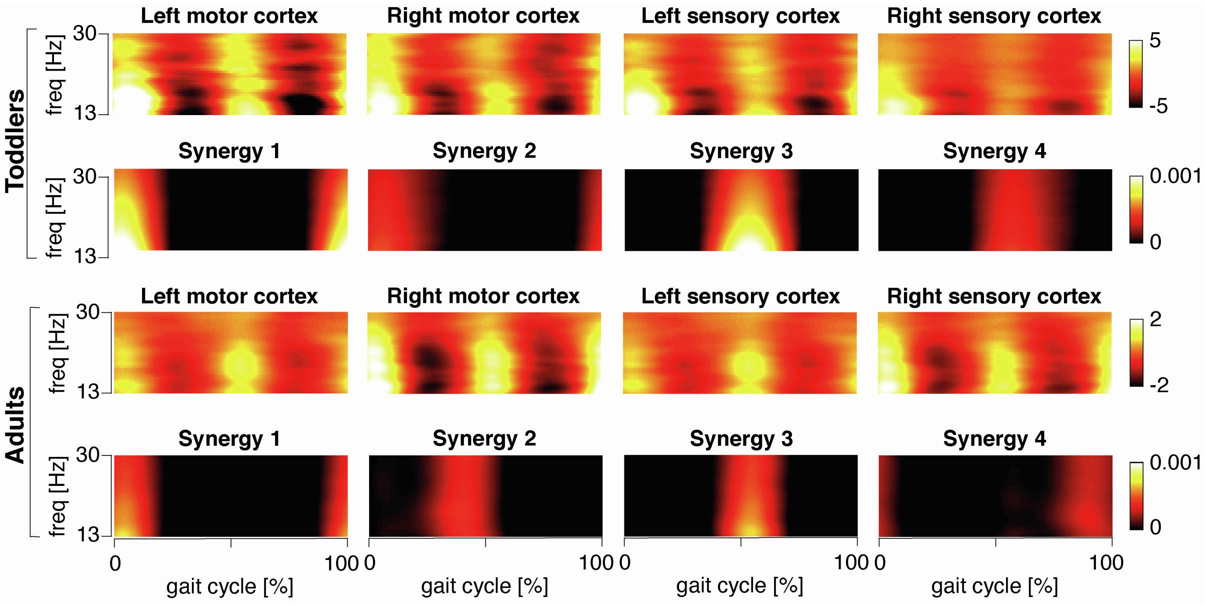

Figure S13. Event-related amplitude modulation of the primary sensorimotor cortices and muscle synergies, related to Figure 3. Amplitude modulation was defined as the instantaneous Hilbert amplitude. The amplitude of the cortical and synergy activity modulates as a function of the gait cycle. The cortical activity shows event-related beta-band [de-]synchronization. Beta-band amplitude of the virtual activation patterns is temporally aligned to the temporal patterns of the synergies. freq: frequency

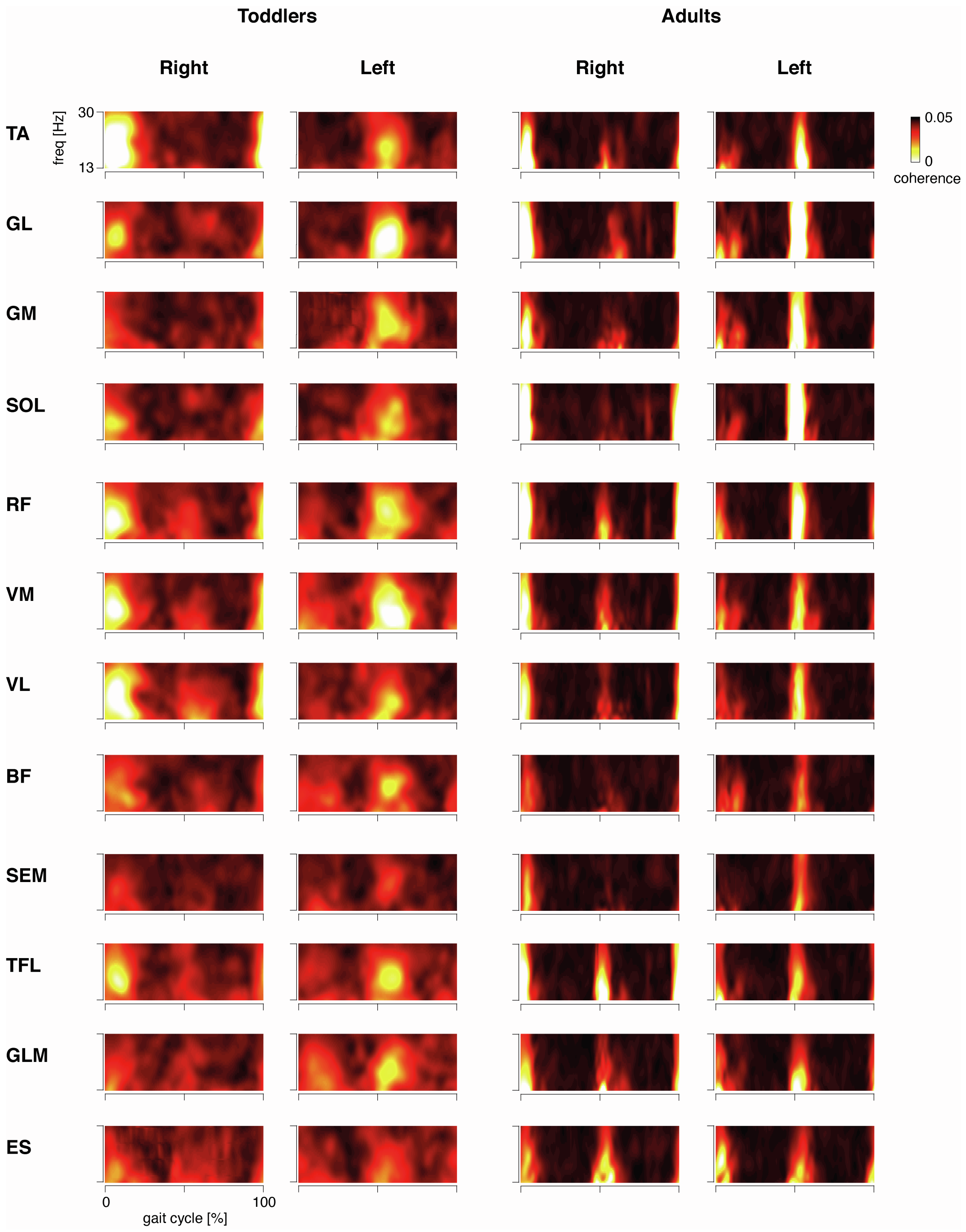

Figure S14. Pairwise cortico-muscular coherence estimates between channel Cz and all individual muscles, related to Figure 3. Gait cycle is defined from right-to-right foot contact. Time-frequency coherences are pooled over participants for every muscular pattern for toddlers and adults. Abbreviations: freq - frequency; TA - tibialis anterior; GL - gastrocnemius lateralis; GM - gastrocnemius medialis; SOL - soleus; RF, rectus femoris; VM - vastus medialis; VL - vastus lateralis; BF - biceps femoris; SEM - semitendinosus; TFL - tensor fasciae latae; GLM - gluteus maximus; ES - erector spinae.

Table S1. Test-statistic characteristics of the cortical muscle synergy representations, related to Figure 2.

	# synergy	atlas area	peak t-val.	RAS [mm]	MNI [mm]	Talairach [mm]
toddlers	S1	left superior parietal gyrus	6.31	[0, -51, 63]	[0, -47, 84]	[0, -42, 79]
	S2	-	-	-	-	-
	S3	left precentral gyrus	5.87	[-5, -26, 62]	[-5, -22, 82]	[-5, -18, 76]
	S4	-	-	-	-	-
adults	S1	left postcentral gyrus; left superior parietal gyrus	4.54	[-23, -35, 52]	[-22, -25, 65]	[-22, -21, 61]
	S2	-	-	-	-	-
	S3	left middle frontal gyrus	4.17	[-31, 26, 37]	[-30, 34, 43]	[-30, 35, 38]
	S4	-	-	-	-	-

Table S2. Mean fraction of negative activations within the virtual temporal components averaged across participants, related to Figure 2.

	toddlers	***adults***
synergy 1	0.0	0.0
synergy 2	0.111	0.071
synergy 3	0.0	0.018
synergy 4	0.100	0.088
